# Classifying chronic pain using ICD-11 and questionnaires—reported characteristics in Japanese patients with chronic pain

**DOI:** 10.3389/fpain.2024.1430870

**Published:** 2024-11-18

**Authors:** Hiroki Igari, Shuichi Aono, Hani M. Bu-Omer, Chie Kishimoto, Aya Nakae, Takahiro Ushida

**Affiliations:** ^1^ Multidisciplinary Pain Center, Aichi Medical University, Nagakute, Japan; ^2^ Department of Software Science, Tamagawa University, Machida, Japan; ^3^ Presence Media Research Group, Hiroshi Ishiguro Laboratories, Deep Interaction Laboratory Group, Advanced Telecommunications Research Institute International (ATR), Kansai Science City, Japan

**Keywords:** ICD-11, chronic pain, Japanese patients, chronic primary pain, chronic secondary pain

## Abstract

**Introduction:**

The new ICD-11 code for chronic pain indicates a direction to divide chronic pain into two categories: chronic secondary pain, which has a clear underlying disease, and chronic primary pain, which is associated with significant emotional distress or functional disability and cannot be explained by another chronic condition. Until now, epidemiological studies have been hampered by the lack of a clear classification, but we believe that this new code system will provide a new perspective on the diagnosis and treatment of chronic pain, and we have begun work on this code system.

**Methods:**

We studied 2,360 patients at Aichi Medical University, the largest pain center in Japan, and asked them to answer questionnaires on pain severity (NRS), pain-related functional impairment (PDAS, Locomo25), quality of life (EQ-5D), and psychological state and pain cognition (HADS, PCS, PSEQ, AIS) while their attending physicians were giving diagnoses according to ICD-11 and the results of the study were used to determine the coding of pain severity.

**Results and discussion:**

The ratio of primary to chronic secondary pain was almost 50%, and the group of patients with MG30.01 classification, which included fibromyalgia, had the highest severity among chronic primary pain. The MG30.01 classification of patients was also found to experience more severe pain compared to other classifications of chronic primary pain patients. The classification of patients with a major psychiatric component was not always clear, and some patients in the secondary category also had a clear psychiatric component, suggesting the need to develop complementary tools to support pain diagnosis.

## 1 Introduction

Like people around the world, many in Japan suffer from chronic pain. A large-scale survey by Japan's Ministry of Health, Labour and Welfare (MHLW) found that between 5% and 10% of Japanese people suffer from chronic conditions such as back pain, headaches, and joint pain (1). Chronic pain can lead to impairment of daily life and a decline in quality of life, prompting many people to visit hospitals. In addition, economic losses from chronic pain in Japan are estimated to be nearly 2 trillion yen, according to an analysis by Inoue et al. (2). In 2010, MHLW compiled “Recommendations for Future Measures to Prevent Chronic Pain” to understand the current situation, including the incidence, types, current responses, and treatment effects of chronic pain. These recommendations aim to elucidate the pathophysiology of intractable pain, develop diagnostic methods and medical systems, and improve quality of life (3). According to a 2010 survey, 22.5% of Japan's adult population, or approximately 23.15 million individuals, experienced chronic pain lasting more than 3 months (4). It is well-recognized that chronic pain is not merely a biological problem but also has psychological and social components (5). Chronic pain is a complex condition that requires multifaceted analysis and diagnosis (6).

The International Classification of Diseases 11th Revision (ICD-11), developed by the World Health Organization, provides a comprehensive and standardized classification system for diseases and health conditions (7). Within this system, chronic pain is categorized into chronic primary pain (MG30.0) and chronic secondary pain (MG30.1 to MG30.Z) (8). Chronic primary pain is a condition in itself, often without an identifiable underlying cause, while chronic secondary pain is associated with an underlying condition such as neuropathy or arthritis (9). The chronic primary pain (MG30.0) entity will also provide a framework to unite conditions that have previously been scattered throughout the ICD and help focus on their commonalities and differences. This distinction is crucial for proper diagnosis and treatment planning.

In Japan, under the leadership of the MHLW, the establishment of multidisciplinary pain centers is being promoted, and currently, more than 30 hospitals have multidisciplinary pain departments (10). However, large-scale implementation studies using ICD-11 have not yet been conducted.

In our study, we utilized the ICD-11 classification to systematically analyze chronic pain conditions. The novelty of our research lies in the application of the ICD-11 codes to a large cohort of Japanese patients, which has not been extensively done before. This study aims to code patients at Japanese multidisciplinary pain centers using ICD-11 and investigate the background characteristics and differences in questionnaire scores for each diagnosis. Additionally, this study aims to provide valuable insights into the characteristics of chronic pain in Japan, as well as to explore the differences between chronic primary widespread pain (MG30.01) and other typers of chronic primary pain, which can inform better clinical practices and policy-making.

## 2 Materials and methods

### 2.1 Participants and procedure

Aichi Medical University Hospital Multidisciplinary Pain Center (11) is an outpatient clinic that accepts both referred patients and in-hospital consultations. As the largest pain center in Japan, it manages 600–700 new pain patients annually. The center specializes in the comprehensive management of all types of chronic pain conditions. The multidisciplinary team is composed of medical professionals, including physicians specialized in orthopedics, anesthesiology, psychiatry, and internal medicine, as well as dentists, nurses, physical therapists, and certified psychologists. The inclusion criteria were chronic pain patients (those experiencing pain for more than three months), patients aged 10 years and older, and patients for whom ICD-11 coding could be performed at clinical conferences. All new patients provided written informed consent at their initial visit. Following this, patients were asked to complete questionnaires using tablet devices. Patients were instructed to fill out all questionnaires under the supervision of trained staff to ensure accuracy and consistency in responses.

Excluding criteria included patients diagnosed acute pain, those whose symptoms had improved by the visit day, and the cases presenting numbness without pain.

### 2.2 ICD-11 coding

The multidisciplinary team at Aichi Medical University Hospital Multidisciplinary Pain Center diagnosed each patient using ICD-11 coding. For each patient, ICD-11 coding was performed using the subcategories (MG30.0 to MG30.Z) of the major category chronic pain (MG30), based on the WHO's ICD-11 website (7) and classification flowchart (8, 9, 12–16). This coding was conducted during clinical case conferences using information obtained from preliminary assessments and physician examinations. In this study, we did not use the new optional ICD-11 extension codes for pain severity. Difficulties encountered with ICD-11 coding in real cases were summarized and discussed at clinical conferences.

### 2.3 Questionnaires

In this study, we collected data on age, gender, height, and weight. Additionally, the following questionnaires were administered.

#### 2.3.1 Numerical rating scale

Patients were asked to indicate the intensity of their pain on the Numerical Rating Scale (NRS). A 11-point scale was used, with 0 representing “no pain” and 10 representing the “most severe pain imaginable”. Patients reported their highest, lowest, and average pain levels over the past 24 h, as well as pain levels when lying down and when moving.

#### 2.3.2 Pain disability assessment scale

To assess pain-related disability of each patient, the Pain Disability Assessment Scale (PDAS) (17), a validated tool measuring impairment daily activities due to pain was used for this study. The PDAS consists of 20 items, each rated on a scale from 0 (no disability) to 10 (total disability), covering various aspects of daily life including personal care, lifting, walking, sitting, standing, sleeping, social life, traveling, and work. The total score is calculated by summing the individual item scores, with higher scores indicating greater disability (18). The reliability of the questionnaires used in this study was assessed by calculating Cronbach's alpha. Based on the current sample, the Cronbach's alpha for PDAS was 0.979.

#### 2.3.3 Hospital anxiety and depression scale

The Hospital Anxiety and Depression Scale (HADS) (19, 20) comprises two subscales, namely HADS for anxiety (HADS-A) and HADS for depression (HADS-D). Both HADS-A and HADS-D consist of seven items; the participants responded to each item on a four-point (0–3) response category, with possible scores ranging from 0 to 21. Anxiety/depression was defined as HADS-A/HADS-D score ≥8. Anxiety severity was defined as follows: 0–7, no anxiety; 8–10, mild anxiety; 11–14, moderate anxiety; and 15–21, severe anxiety. Depression severity was defined as follows: 0–7, no depression; 8–10, mild depression; 11–14, moderate depression; and 15–21, severe depression (21). The Cronbach's alpha for HADS was 0.884, based on the current sample.

#### 2.3.4 Pain catastrophizing scale

Pain catastrophizing refers to the cognitive and emotional responses to anticipated or actual painm characterized by negative thinking patterns such as rumination, magnification, and feelings of helplessness. In this study, the Pain Catastrophizing Scale (PCS) (22) was utilized to assess these reponses. The PCS comprises 13 items that measure thoughts and feelings related to pain across three dimensions: rumination, magnification, and helplessness. Each item is scored on a 5-point Likert scale ranging from 0 (“not at all”) to 4 (“all the time”), with the total possible score ranging from 0 to 52. Higher scores indicate greater levels of pain catastrophizing, a psychological condition associated with poorer pain outcomes and heightened emotional distress. The PCS is particularly advantageous in clinical research due to its strong psychometric properties, including high internal consistency and established validity for assessing maladaptive pain responses in diverse populations (23). The Cronbach's alpha for PCS was 0.889, based on the current sample.

#### 2.3.5 Euroqol 5 dimensions

The EuroQol 5 Dimensions (EQ-5D) (24) index values were recorded to assess the quality of life of patients. In the current study, we employed the EQ-5D, a standardized instrument for measuring general quality of life. The EQ-5D encompasses five dimensions: mobility, self-care, usual activities, pain/discomfort, and anxiety/depression. Each dimension has three levels: no problems, some problems, and extreme problems. Participants provide responses based on their current health state, which are then converted into a single summary index by applying a formula that incorporates weights derived from population studies. The use of EQ-5D in our study facilitates the assessment of health-related quality of life (HRQoL) across different patient groups, providing a quantitative measure that is easily comparable across contexts and studies (25). The Cronbach's alpha for EQ-5D was 0.788, based on the current sample.

#### 2.3.6 Pain self-efficacy questionnaire

To evaluate participants' beliefs about their ability to perform tasks despite their pain, the Pain Self-Efficacy Questionnaire (PSEQ) (26) was employed in this study. The PSEQ is a widely validated instrument consisting of 10 items that assess various aspects of self-efficacy in pain management. Each item is scored on a 7-point Likert scale, ranging from 0 (“not at all confident”) to 6 (“completely confident”), with the total score ranging from 0 to 60. Higher scores indicate greater pain self-efficacy, suggesting a stronger belief in the individual's capacity to manage daily activities despite experiencing pain (27). The Cronbach's alpha for PSEQ was 0.949, based on the current sample.

#### 2.3.7 Athens insomnia scale

In this study, the Athens Insomnia Scale (AIS) (28) was utilized to assess insomnia severity among participants. The AIS is a brief, self-administered psychometric instrument developed to quantify sleep difficulty based on the ICD-10 criteria for insomnia. It consists of 8 items that evaluate the quality and efficiency of sleep over the past month. Each item is scored on a scale from 0 (no problem) to 3 (serious problem), with the total score ranging from 0 to 24. Higher scores indicate more severe insomnia symptoms (29). The Cronbach's alpha for AIS was 0.841, based on the current sample.

#### 2.3.8 Geriatric locomotive function scale

The 25-question Geriatric Locomotive Function Scale (Locomo25) (30, 31) was utilized to assess the risk of locomotive syndrome. This scale comprises 25 questions evaluating musculoskeletal disorders, mobility difficulties, daily living challenges, and pain. Each question helps identify individuals at high risk of requiring care unless intervened medically. The scale's reliability and validity have been confirmed through extensive research. The Cronbach's alpha for Locomo25 was 0.962, based on the current sample.

### 2.4 Statistical analyses

We compared the distribution of ICD-11 coding and questionnaire scores between chronic primary pain (MG30.01) and chronic secondary pain (MG30.1 to MG30.Z) using descriptive statistics. Additionally, within the chronic primary pain category, we performed a separate analysis comparing chronic primary widespread pain (MG30.01) with the other subcategories (MG30.00 and MG30.02 to MG30.0Z). Differences in all questionnaire scores across patient groups were analyzed using the Tukey–Kramer test (32, 33), which allows for pairwise comparisons while controlling the family-wise error rate. Statistical significance set at *p* < 0.05.

Differences by age group and sex were analyzed using the Wald *χ*^2^ test (34), which is suitable for categorical data analysis and tests the association between categorical variables. Statistical significance set at *p* < 0.05. For age groups, distributions were examined by dividing participants into three categories: under 18 years, 18–64 years, and over 65 years. Effect sizes were also calculated to assess the magnitude of the differences observed. Eta squared (*η*^2^) was used to calculate the effect size for questionnaire scores (35). Cramer's *V* was used to evaluate the effect size for categorical data (36).

All statistical analyses were conducted using JMP statistical software version 17. The rationale for choosing these methods was to accurately compare mean differences among multiple groups (Tukey–Kramer test) and to assess the associations between categorical variables (Wald *χ*^2^ test). The *p*-values and effect sizes were used to identify and quantify statistically significant differences.

## 3 Results

### 3.1 Sample characteristics

Participants included 2,586 male and female patients enrolled at the Aichi Medical University Hospital Multidisciplinary Pain Center in Japan from 2016 to 2021. After careful consideration of the inclusion criteria, data from 2,360 patients were included in this study. A total of 171 datasets were excluded based on the exclusion criteria: 16 patients were excluded due to acute pain, 77 patients were excluded for experiencing numbness without pain, and 78 were excluded as deemed inappropriate by their attending physicians.

The study included a total of 2,360 patients with an average age of 55.6 years. The distribution of age groups was as follows: 105 patients (4.4%) were in the younger group (under 18 years), 1,337 patients (56.7%) were in the adult group (18–64 years), and 918 patients (38.9%) were in the older group (over 65 years). The average height of the patients was 160.4 cm, and the average weight was 57.9 kg. Regarding gender distribution, there were 1,418 female patients, accounting for 60.1% of patients. These characteristics were shown in Table 2A. Additionally, the questionnaire scores were shown in Table 2B.

### 3.2 The distribution of ICD-11 coding in all patients

As shown in Table 1, 1,178 patients (49.9%) were categorized under chronic primary pain category (MG30.0), whereas 1,182 patients (50.1%) were included chronic secondary pain category (MG30.1 to MG30.Z). Within the chronic secondary pain category, secondary musculoskeletal pain (MG30.3) was the most common reason, accounting for 443 patients (18.8%), followed by chronic neuropathic pain [MG30.5, 415 patients (17.7%)] and Chronic postsurgical or post-traumatic pain [MG30.2, 261 patients (11.0%)].

**Table 1:** Breakdown of visited chronic pain patients in Japanese study group.

ICD-11	Patients [*n* (%)]
MG30.00 Chronic primary visceral pain	81 (3.43%)
MG30.01 Chronic widespread pain	296 (12.54%)
MG30.02 Chronic primary musculoskeletal pain	443 (18.77%)
MG30.03 Chronic primary headache or orofacial pain	324 (13.73%)
MG30.04 Complex regional pain syndrome	17 (0.72%)
MG30.0Y Other specified chronic primary pain	9 (0.38%)
MG30.0Z Chronic primary pain, unspecified	8 (0.34%)
MG30.1 Chronic cancer related pain	20 (0.85%)
MG30.2 Chronic postsurgical or post traumatic pain	261 (11.03%)
MG30.3 Chronic secondary musculoskeletal pain	431 (18.26%)
MG30.4 Chronic secondary visceral pain	11 (0.46%)
MG30.5 Chronic neuropathic pain	415 (17.67%)
MG306 Chronic secondary headache or orofacial pain	44 (1.86%)

**Figure 1:**
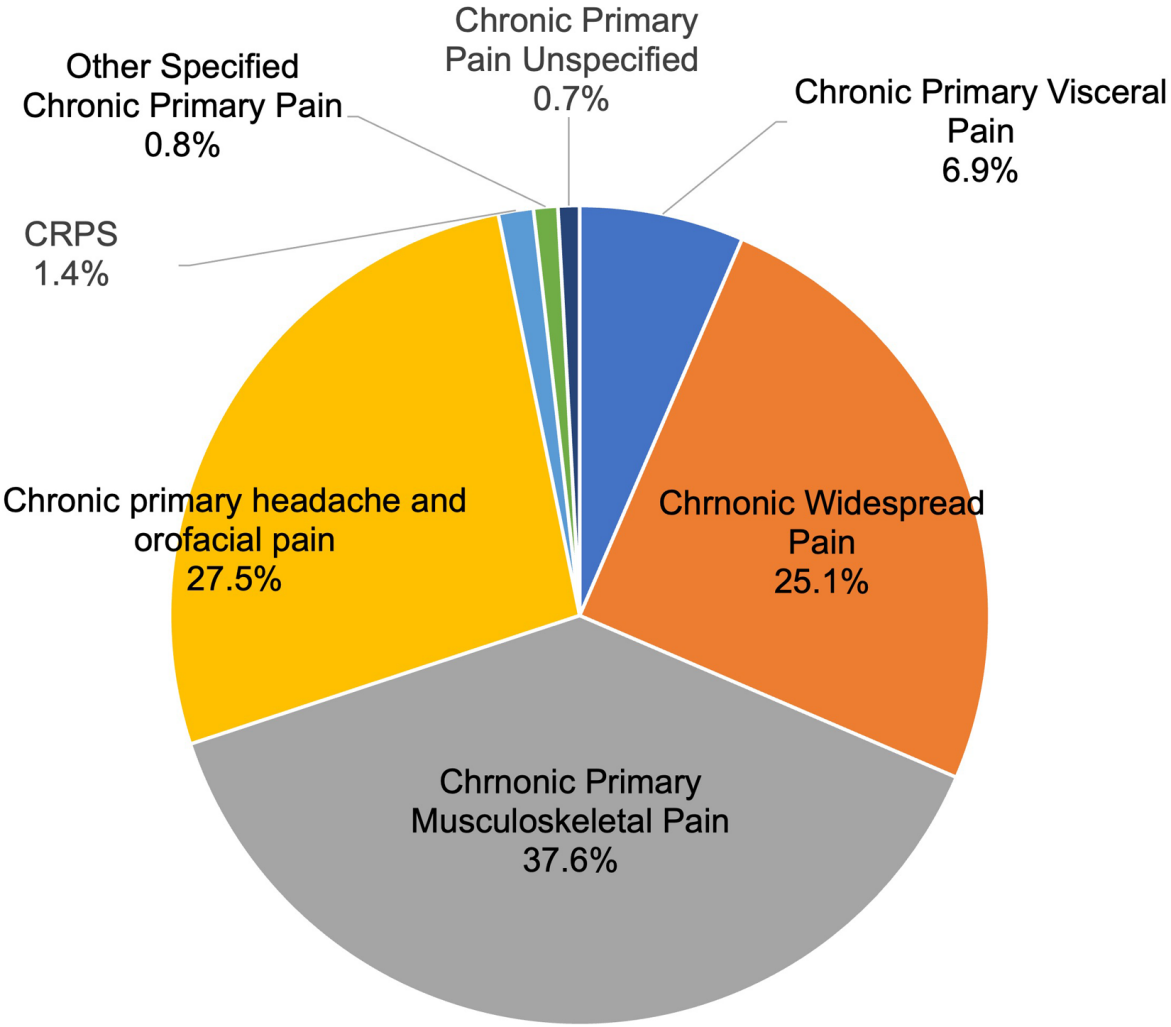
Breakdown of chronic primary pain cases.

As shown in Figure 1, within the chronic primary pain category (MG30.0, 1,178 patients), the top three categories were chronic primary musculoskeletal pain [MG30.02, 443 patients (37.6%)], chronic primary headache and orofacial pain [MG30.03, 324 patients (27.5%)], then chronic widespread pain [MG30.01, 296 patients (25.1%)].

**Figure 2:**
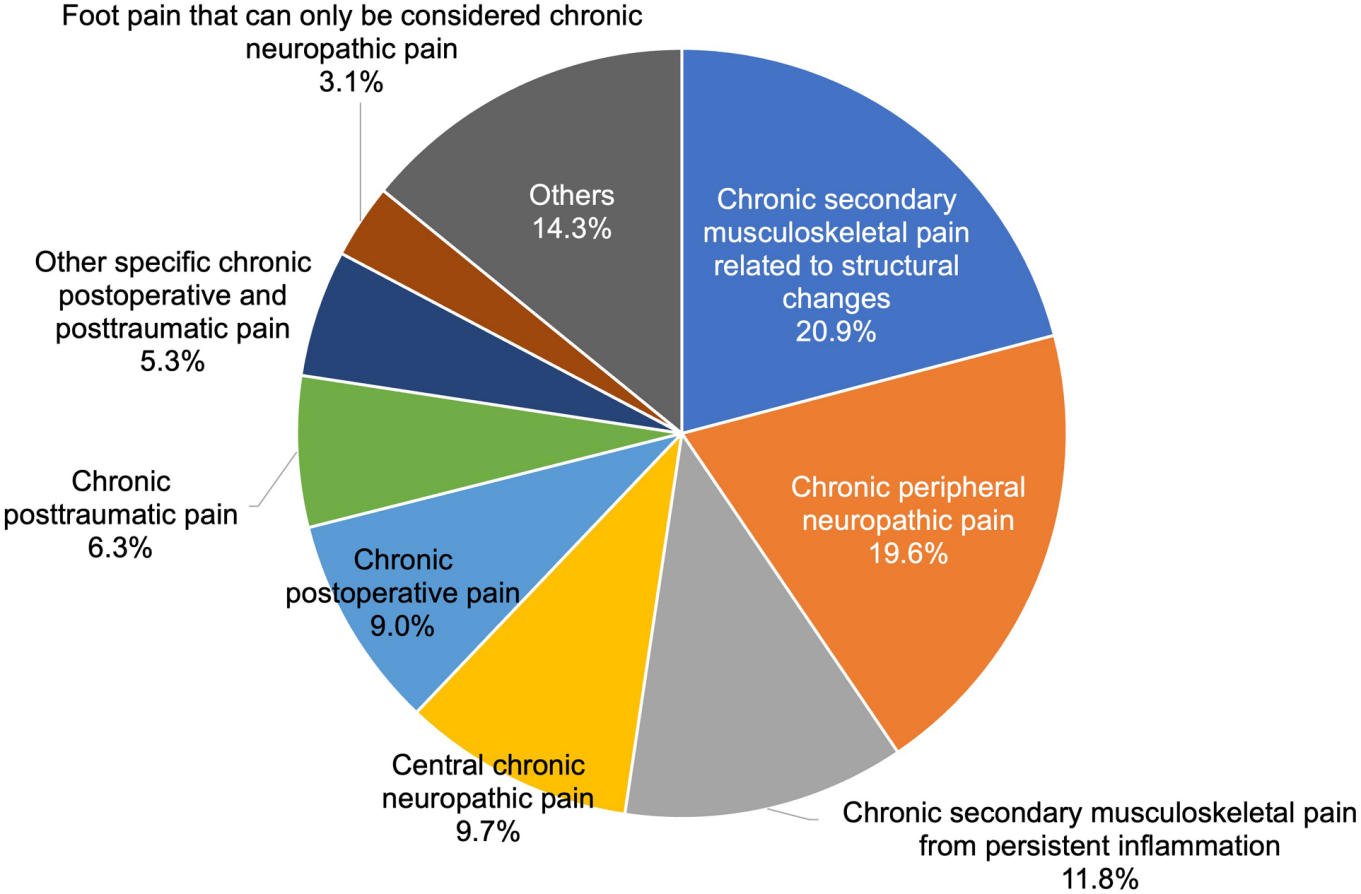
Breakdown of chronic secondary pain cases.

As shown in Figure 2, within chronic secondary pain category (MG30.1 to MG30.Z, 1,182 patients), the top three categories were chronic secondary musculoskeletal pain related to structural changes [MG30.31, 247 patients (20.9%)], chronic peripheral neuropathic pain [MG30.51, 232 patients (19.6%)], then chronic secondary musculoskeletal pain from persistent inflammation [MG30.30, 140 patients (11.8%)].

### 3.3 The age differences of distribution of ICD-11

**Figure 3:**
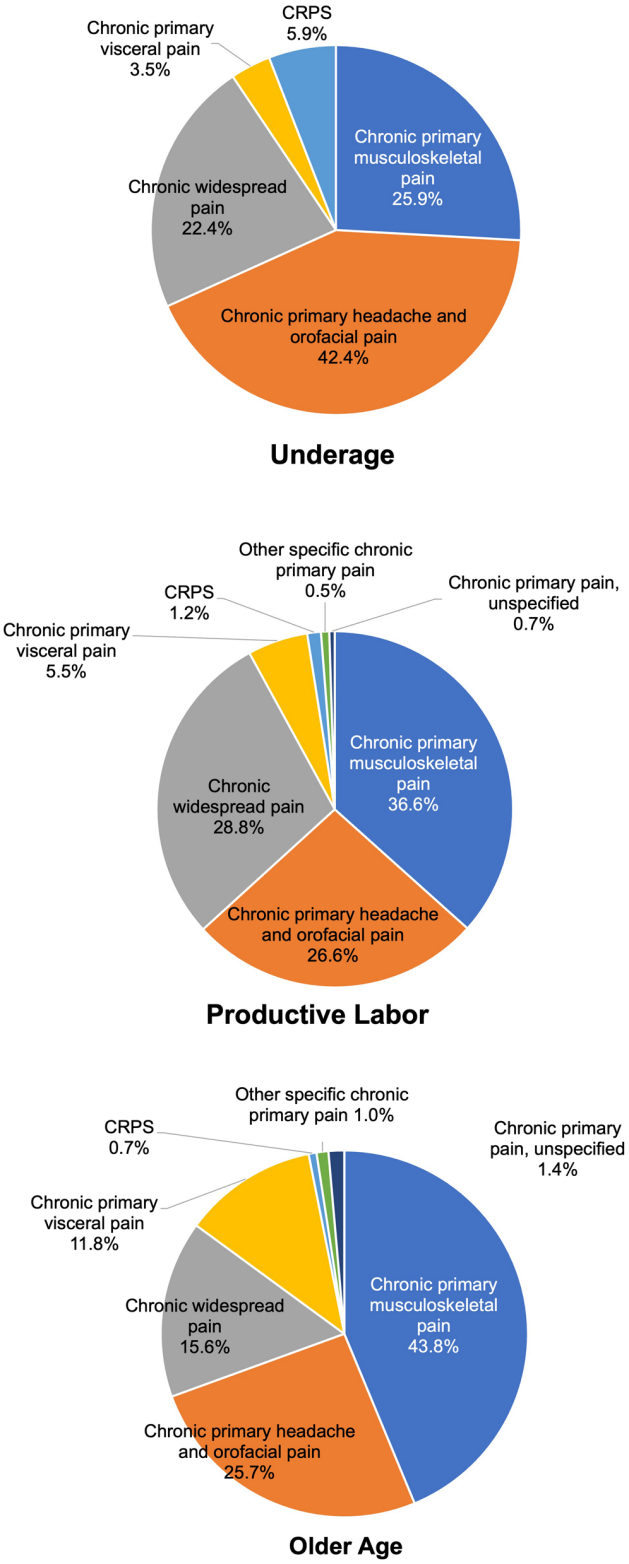
Distribution of chronic primary pain by age group.

As shown in Figure 3, within the chronic primary pain category (MG30.0), differences by age group were examined. In the older age group (over 65 years, 288 patients), the top three categories were chronic primary musculoskeletal pain [MG30.02, 126 patients (43.8%)], chronic primary headache and orofacial pain [MG30.03, 74 patients (25.7%)], then chronic widespread pain [MG30.01, 45 patients (15.6%)]. Conversely, in the productive labor age group (18–64 years, 805 patients), the top three categories were chronic primary musculoskeletal pain [MG30.02, 295 patients (36.6%)], chronic primary headache and orofacial pain [MG30.03, 214 patients (26.6%)], then chronic widespread pain [MG30.01, 232 patients (28.8%)]. Chronic primary musculoskeletal pain (MG30.02) was more prevalent in the older age group than in the productive labor age group, while chronic widespread pain (MG30.01) was more common in the productive labor age group. In the younger age group (under 18 years, 85 patients), the distribution was as follows: chronic primary musculoskeletal pain (MG30.02) accounted for 22 patients (25.9%), chronic primary headache and orofacial pain (MG30.03) for 36 patients (42.4%), chronic widespread pain (MG30.01) for 19 patients (22.4%), and complex regional pain syndrome (CRPS) (8D8A.0) for 5 patients (5.9%).

**Figure 4:**
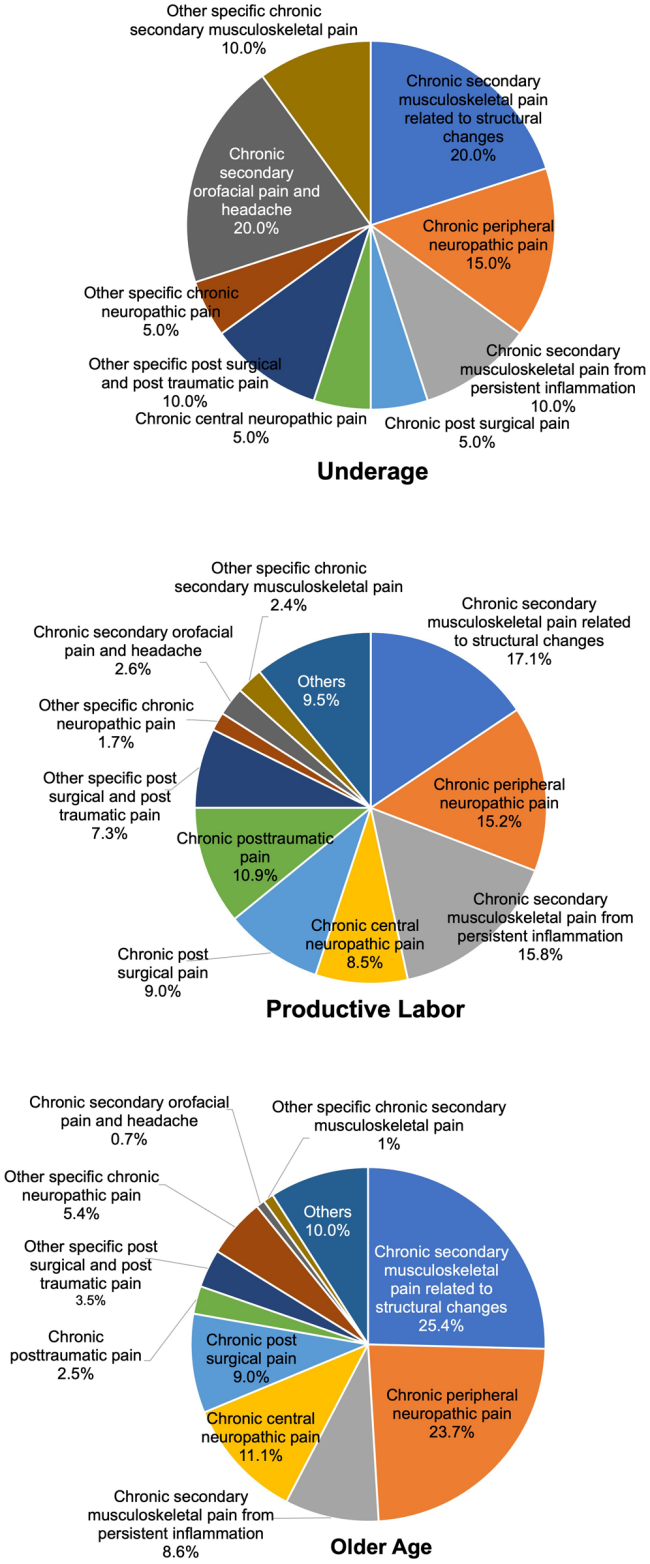
Distribution of top 10 chronic secondary pain by age group.

As shown in Figure 4, within the chronic secondary pain category (MG30.1 to MG30.Z), differences by aging group were examined. The top three categories in the older age group (over 65 years, 630 patients) were chronic secondary musculoskeletal pain associated with structural changes (MG30.31, 91 patients (25.4%), chronic peripheral neuropathic pain [MG30.51, 149 patients (23.7%)], then chronic central neuropathic pain [MG30.50, 70 patients (11.1%)]. On the other hand, in the productive labor age group (18–64 years, 532 patients), the top three categories were chronic secondary musculoskeletal pain associated with structural changes [MG30.31, 91 patients (17.1%)], chronic peripheral neuropathic pain [MG30.51, 81 patients (15.2%)], then chronic secondary musculoskeletal pain from persistent inflammation [MG30.30, 84 patients (15.8%)]. In the younger age group (under 18 years, 20 patients), the top three categories were chronic secondary musculoskeletal pain associated with structural changes [MG30.31, 4 patients (20.0%)], chronic secondary orofacial pain and headache [MG30.6, 4 patients (20.0%)], then chronic peripheral neuropathic pain [MG30.51, 3 patients (15.0%)].

### 3.4 The different features between the chronic primary pain and the chronic secondary pain

As shown in Table 2A, average of the patients' age in the chronic secondary pain group was significantly more than in the chronic primary pain group [average ± SD = 49.2 ± 18.8, 61.9 ± 16.7; *p* < .0001, with a medium effect size (*η*^2^ = 0.112)]. About the sex distribution, the chronic primary pain group has higher the rate of female than in the chronic secondary pain group (*p* = 0.0018), but the effect size was negligible (Tables 2A,B).

**Table 2A:** The characteristics of the patients and the difference between primary chronic pain and the secondary chronic pain.

		Total (*n* = 2,360)	Primary (*n* = 1,178; 49.9%)	Secondary (*n* = 1,182; 50.1%)	*p* value	Effect size (*η*^2^)	Effect size (Cramer's V)
Age average		55.6 ± 18.9	49.2 ± 18.8	61.9 ± 16.7	<0.0001	0.112^†^	
Height, cm		160.4 ± 8.9	160.9 ± 8.7	160.0 ± 8.9	0.0165	<0.01	
Weight, kg		57.9 ± 12.0	57.4 ± 12.9	58.3 ± 12.0	0.1139	<0.01	
Age group	Younger (under 18 years), *n* (%)	105 (4.4%)	85 (7.2%)	20 (1.7%)	<0.0001		0.134*
	Adult (18–64 years), *n* (%)	1,337 (56.7%)	805 (68.3%)	532 (45.0%)			0.235*
	Older (over 65 years), *n* (%)	918 (38.9%)	288 (24.4%)	630 (53.3%)			0.295*
Sex (female), *n* (%)		1,418 (60.1%)	745 (63.2%)	673 (56.9%)	0.0018		<0.1

Note: Value given in Average ± Standard Deviation, *η*^2^ values with asterisk (*) indicating small effect size (0.01–0.06) and dagger (†) indicating medium effect size (0.06–0.14), Cramer's *V* values with asterisk (*) indicating small effect size (0.1–0.3).

**Table 2B:** The characteristics of the patients and the difference between primary chronic pain and the secondary chronic pain.

	Total	Primary	Secondary	*p* value	Effect size (*η*^2^)
NRS highest	6.61 ± 2.42	6.41 ± 2.50	6.81 ± 2.33	<0.0001	<0.01
NRS lowest	3.39 ± 2.51	3.22 ± 2.49	3.57 ± 2.53	0.0008	<0.01
NRS average	5.78 ± 2.18	5.66 ± 2.22	5.89 ± 2.12	0.0096	<0.01
NRS When lying down	4.51 ± 2.82	4.54 ± 2.84	4.49 ± 2.80	0.7254	<0.01
NRS When moving	6.11 ± 2.67	5.94 ± 2.73	6.29 ± 2.59	0.0015	<0.01
PDAS (Pain Disability Assessment Scale)	23.34 ± 12.99	21.30 ± 12.59	25.38 ± 13.06	<0.0001	0.024*
HADS (Hospital Anxiety and Depression Scale)	16.10 ± 8.36	16.63 ± 8.67	15.56 ± 8.02	0.0019	<0.01
HADS Anxiety	7.85 ± 4.39	8.22 ± 4.51	7.49 ± 4.23	<0.0001	<0.01
HADS Depression	8.24 ± 4.71	8.42 ± 4.83	8.07 ± 4.59	0.0731	<0.01
PCS (Pain Catastrophizing Scale)	35.11 ± 9.77	34.86 ± 9.67	35.36 ± 9.87	0.2200	<0.01
PCS rumination	12.82 ± 2.97	12.75 ± 2.99	12.89 ± 2.95	0.2541	<0.01
PCS magnification	6.93 ± 3.03	6.87 ± 2.99	6.99 ± 3.08	0.3412	<0.01
PCS helplessnes	15.36 ± 5.10	15.24 ± 5.01	15.48 ± 5.18	0.2625	<0.01
EQ-5D (Health Questionnaire)	0.565 ± 0.201	0.582 ± 0.197	0.548 ± 0.204	<0.0001	<0.01
PSEQ (Pain Self-Efficacy Questionnaire)	25.81 ± 14.46	24.62 ± 13.96	27.00 ± 14.85	<0.0001	<0.01
AIS (Athene Insomnia Scale)	8.34 ± 8.34	8.44 ± 4.91	8.24 ± 4.83	0.3321	<0.01
Locomo25 (25-question Geriatric Locomotive Function Scale)	33.43 ± 22.72	29.42 ± 21.16	37.43 ± 23.50	<0.0001	0.031*

Note: Value given in Average ± Standard Deviation, *η*^2^ values with asterisk (*) indicating small effect size (0.01–0.06).

Analysis of PDAS scores indicated statistically significant greater disability in patients with chronic secondary pain (25.38 ± 13.06) compared to those with chronic primary pain (21.30 ± 12.59) [*p* < 0.0001, with a small effect size (*η*^2^ = 0.024)].

The average HADS-Anxiety and HADS-Total scores were significantly higher in the chronic primary pain group than in the chronic secondary pain group (Anxiety; 8.22 ± 4.51, 7.49 ± 4.23, *p* < 0.0001: Total; 16.63 ± 8.67, 15.56 ± 8.02, *p* = 0.0019), but the effect size was negligible.

Analysis of the Pain PCS scores did not reveal significant differences in pain catastrophizing between patients suffering from chronic primary and secondary pains. Both groups of patients with chronic pain were found to exhibit high pain catastrophizing tendencies.

The EQ-5D results indicated significant differences in health-related quality of life among the patient groups. Notably, patients with chronic secondary pain conditions reported lower EQ-5D index values (0.548 ± 0.204), reflecting poorer health status compared to those with primary pain conditions (0.582 ± 0.197) (*p* < 0.0001), but the effect size was negligible.

Results from the PSEQ revealed significant differences in self-efficacy among the study groups. Patients chronic primary pain conditions reported higher PSEQ scores (24.62 ± 13.96) compared to those experiencing high-intensity chronic pain (27.00 ± 14.85) (*p* < 0.0001), but the effect size was negligible.

The AIS results did not show significant differences in sleep disturbance between primary and chronic secondary pain patients, however, average scores in both groups were over 8 with the possibility of insomnia (8.44 ± 4.91 in primary and 8.24 ± 4.83 in secondary).

Analysis of the Locomo25 scores among patients with primary and chronic secondary pain conditions revealed significant differences in locomotive function. Patients with chronic secondary pain reported higher Locomo25 scores (37.43 ± 23.50), indicative of more severe mobility restrictions and greater discomfort, compared to those with chronic primary pain (29.42 ± 21.16) [*p* < 0.0001, with a small effect size (*η*^2^ = 0.031)]. This indicates a higher incidence and severity of locomotive syndrome in patients with chronic secondary pain, highlighting the scale's utility in identifying high-risk individuals.

### 3.5 The different features between the MG30.01 and other chronic primary pain

As shown in Table 3A, the average age of the patients showed no difference, however, when divided into three groups, there was a significant difference in the age group category [*p* < 0.0001, with a small effect size (Cramer's *V* = 0.124)]. About the sex distribution, the chronic primary pain has more the rate of female than in the chronic secondary pain (*p* = 0.0034), but the effect size was negligible (Tables 3A,B).

**Table 3A:** The differences between MG30.01 (chronic primary widespread pain) and other types of chronic primary pain.

		MG30.01 (*N* = 296; 25.1%)	Others (*N* = 882; 74.9%)	*p* value	Effect size (*η*^2^)	Effect size (Cramer's V)
Age Average		46.8 ± 17.0	50.1 ± 19.3	0.0097	<0.01	
Height, cm		160.3 ± 8.2	161.1 ± 9.4	0.1952	<0.01	
Weight, kg		57.4 ± 12.9	57.4 ± 13.3	0.9268	<0.01	
Age Group	Younger (under 18 years), *n* (%)	19 (6.4%)	66 (7.5%)	<0.0001		<0.1
	Adult (18–64 years), *n* (%)	232 (78.4%)	573 (65.0%)			0.125*
	Older (over 65 years), *n* (%)	45 (15.2%)	243 (27.6%)			0.124*
Sex (female), *n* (%)		208 (70.3%)	537 (60.9%)	0.0034		<0.1

Note: Value given in Average ± Standard Deviation, *η*^2^ values with asterisk (*) indicating small effect size (0.01–0.06) and dagger (†) indicating medium effect size (0.06–0.14), Cramer's *V* values with asterisk (*) indicating small effect size (0.1–0.3).

**Table 3B:** The differences between MG30.01 (chronic primary widespread pain) and other types of chronic primary pain.

	MG30.01	Others	*p* value	Effect size (*η*^2^)
NRS highest	7.11 ± 2.10	6.18 ± 2.58	<0.0001	0.026*
NRS lowest	3.77 ± 2.54	3.03 ± 2.44	<0.0001	0.016*
NRS average	6.18 ± 2.06	5.49 ± 2.25	<0.0001	0.018*
NRS When lying down	5.35 ± 2.73	4.26 ± 2.82	<0.0001	0.027*
NRS When moving	6.80 ± 2.40	5.65 ± 2.78	<0.0001	0.033*
PDAS (Pain Disability Assessment Scale)	27.31 ± 12.30	19.28 ± 12.04	<0.0001	0.076^†^
HADS (Hospital Anxiety and Depression Scale)	19.52 ± 8.56	15.66 ± 8.48	<0.0001	0.037*
HADS Anxiety	9.64 ± 4.53	7.74 ± 4.40	<0.0001	0.033*
HADS Depression	9.88 ± 4.79	7.93 ± 4.74	<0.0001	0.030*
PCS (Pain Catastrophizing Scale)	36.57 ± 9.78	34.29 ± 9.57	0.0005	0.010*
PCS rumination	12.90 ± 3.02	12.71 ± 2.97	0.3440	<0.01
PCS magnification	7.57 ± 2.98	6.64 ± 2.95	<0.0001	0.018*
PCS helplessnes	16.11 ± 5.03	14.95 ± 4.97	0.0006	0.010*
EQ-5D (Health Questionnaire)	0.499 ± 0.200	0.610 ± 0.188	<0.0001	0.059*
PSEQ (Pain Self-Efficacy Questionnaire)	21.55 ± 13.43	25.64 ± 13.98	<0.0001	0.016*
AIS (Athene Insomnia Scale)	10.43 ± 5.08	7.77 ± 4.65	<0.0001	0.055*
Locomo25 (25-question Geriatric Locomotive Function Scale)	41.87 ± 22.40	25.24 ± 18.98	<0.0001	0.116^†^

Note: Value given in Average ± Standard Deviation, *η*^2^ values with asterisk (*) indicating small effect size (0.01–0.06) and dagger (†) indicating medium effect size (0.06–0.14).

In the chronic primary pain category, PDAS also showed a significantly higher disability in patients with chronic primary widespread pain (MG30.01) compared to other types of chronic primary pain [27.31 ± 12.30, 19.28 ± 12.04, respectively, and *p* < 0.0001, with a medium effect size (*η*^2^ = 0.076)].

The average HADS-Anxiety, HADS-Depression and HADS-Total scores were significantly higher in the chronic primary widespread pain group (MG30.01) than in other chronic primary pain groups [Anxiety; 9.64 ± 4.53, 7.74 ± 4.40, *p* < 0.0001, with a small effect size (*η*^2^ = 0.033)]: Depression; 9.88 ± 4.79, 7.93 ± 4.74, *p* < 0.0001, with a small effect size (*η*^2^ = 0.030)): Total; 19.52 ± 8.56, 15.66 ± 8.48, *p* < 0.0001, with a small effect size (*η*^2^ = 0.037)). The patients in the chronic primary widespread pain (MG30.01) have more severe depression and anxiety than other types of chronic primary pain.

Although there was no differences in overall PCS scores between primary and chronic secondary pain groups, patients diagnosed with chronic primary widespread pain (MG30.01), reported statistically higher PCS magnification and helplessness sub-scores (7.57 ± 2.98 and 16.11 ± 5.03, respectively) compared to patients with other chronic primary pain (6.64 ± 2.95 and 14.95 ± 4.97, respectively, *p* < 0.0001, with a small effect size (*η*^2^ = 0.018) and *p* = 0.0006 with a small effect size (*η*^2^ = 0.010), respectively). This indicates a more pronounced cognitive and emotional response to chronic primary widespread pain.

Within patients with chronic primary pain populations, patients with chronic primary widespread pain (MG30.01) demonstrated significantly lower EQ-5D index values compared to other chronic primary pain types [0.499 ± 0.200, 0.610 ± 0.188, respectively; *p* < 0.0001, with a small effect size (*η*^2^ = 0.059)]. This underscores the substantial impact of pain severity on overall quality of life.

Results from the PSEQ underscored significant differences in self-efficacy across the patient groups. Patients with chronic primary widespread pain (MG30.01) exhibited lower PSEQ scores (21.55 ± 13.43), indicating less confidence in managing daily activities despite their pain, compared to other chronic primary pain conditions (25.64 ± 13.98) [*p* < 0.0001, with a small effect size (*η*^2^ = 0.016)].

Within the chronic primary pain categories, patients with chronic primary widespread pain (MG30.01) reported significantly higher AIS scores, suggesting a possible diagnosis of insomnia (10.43 ± 5.08) compared to other chronic primary pain conditions (7.77 ± 4.65) [*p* < 0.0001, with a small effect size (*η*^2^ = 0.055)].

Comparing the chronic primary pain category, patients suffering from chronic primary widespread pain (MG30.01) also reported significantly higher Locomo25 scores (41.87 ± 22.40) compared to other patients in the category (25.24 ± 18.98) [*p* < 0.0001, with a medium effect size (*η*^2^ = 0.116)].

## 4 Discussion

This study is the first to characterize and analyze chronic pain patients by applying ICD-11 at Japan's largest interdisciplinary pain treatment center. We discussed the differences between chronic primary pain and chronic secondary pain according to the ICD-11, the characteristics within chronic primary pain, and the challenges of ICD-11 coding.

### 4.1 The differences between a primary and a chronic secondary pain

Chronic primary pain, even with organic elements, refers to pain that cannot be fully explained by these elements alone (37). Conditions such as fibromyalgia, nonspecific lower back pain, migraine, and irritable bowel syndrome are considered typical examples, while conditions like nonspecific lower back pain and migraine are often included under chronic primary pain when they lack a clear organic cause (38). These conditions are presumed to result from changes in nerve functionality, such as central sensitization (39). Therefore, even if the pain is located in the same area, it is presumed that chronic primary pain has clinical features that differ from those of chronic secondary pain (40).

The results indicated that patients classified with chronic primary pain were statistically significantly younger and more likely to be female. This aligns with previous reports suggesting that patients with pain modulation issues, as defined by the IASP and with no clear cause, were predominantly female and younger (Table 2A) (41). Additionally, the proportion of chronic primary pain increased as the age groups became younger, further supporting the notion that chronic primary pain is more prevalent in younger populations.

In both groups, we examined the stronger or weaker outliers in the questionnaire results. Subjective pain intensity using NRS, PDAS, and Locomo25 were higher in the chronic secondary pain group. PDAS and Locomo25 had small effect sizes. The majority of patients with chronic secondary pain suffered from musculoskeletal conditions. Actually, their actual problem related to their physical body was considered to affect badly to their PDAS and Locomo25 (42).

On the other hands, HADS scores, especially for anxiety, were higher in chronic primary pain patients with the lower score of PSEQ. These results may be reflected that the symptoms of patients with chronic primary pain may be considered to be evoked by more emotional factors, such as stress (43).

EQ-5D scores were extremely low in both groups compared to healthy individuals and general patients, but they were lower in the chronic secondary pain group. This suggests that chronic secondary pain may have a greater impact on quality of life than chronic primary pain (44). Based on these results, the structural and pathological conditions in chronic secondary pain likely contribute more directly to functional limitations and reduced quality of life. In contrast, while chronic primary pain is significantly influenced by emotional distress, it may not always manifest as severe physical disability, which could explain the relatively lower PDAS and Locomo25 scores in this group. However, since significant effect sizes were not detected for questionnaires other than PDAS and Locomo25, further validation with a larger sample size is necessary.

### 4.2 The differences between widespread type of a chronic primary pain and other types of chronic primary pain

Secondly, we specifically examined the differences between MG30.01 and other types of chronic primary pain with pervasive symptoms. It was found that there was no significant difference in age, but the age distribution was skewed. Both groups were more likely to be female, but a greater female predominance was observed among patients classified with MG30.01. The predominance of female patients and the associated body weight are consistent with previous reports (40).

Patients with MG30.01 (chronic primary widespread pain, including fibromyalgia), had worse outcomes on almost all measures compared to other chronic primary pain categories.

In patients with MG30.01, the NRS scores were high across all queried situations, and both the PDAS and Locomo25 scores were extremely high, despite the absence of a musculoskeletal cause. The marked trend in the age group, which is typically not associated with physical causes, reflects the characteristics of this disease, where psychological factors contribute significantly to physical activity difficulties (45).

HADS scores suggest a subset of patients with pathological anxiety and depression, with scores close to 10 for both conditions. chronic pain patients often experience high levels of anxiety and depression, which can exacerbate their pain perception and reduce their ability to cope with pain (46). These psychological factors might contribute to the higher HADS scores observed in MG30.01 patients​.

PCS scores were also higher than those for other chronic primary pain conditions, suggesting a greater tendency toward catastrophize. Catastrophizing is known to amplify pain perception and contribute to increased disability in chronic pain patients (22). This could explain why MG30.01 patients report higher PCS scores, as they might be more prone to negative thought patterns about their pain (47).

AIS scores were also significantly higher, suggesting persistent conditions that interfere with recovery. Sleep problems are common in chronic pain conditions and can hinder recovery by exacerbating pain sensitivity and reducing pain tolerance (48). Persistent sleep issues in MG30.01 patients could lead to poorer recovery outcomes, reflected in the higher AIS scores.

The PSEQ scores were lower than those for other chronic primary pain conditions, consistent with lower self-efficacy scores in patients with intractable chronic pain. Self-efficacy is crucial for effective pain management and coping strategies (49). Lower self-efficacy has been associated with higher pain intensity and greater disability in chronic pain patients (50). This could explain the lower PSEQ scores in MG30.01 patients, as they might feel less capable of managing their pain effectively.

The EQ-5D scores were also lower than those for other types of chronic primary pain, clearly indicating that MG30.01 has a more detrimental impact on quality of life. Chronic pain significantly affects various aspects of daily living, leading to reduced physical and social functioning (51). The more severe impact on quality of life in MG30.01 patients could be due to the combined effects of high pain intensity, psychological distress, and reduced self-efficacy.

These findings suggest that MG30.01, in particular, represents a unique pathology among those classified as having chronic primary pain, and that patients in this category, many of whom are diagnosed with fibromyalgia, require more focused attention (52).

### 4.3 The difficult points to apply ICD-11 code to each patient

One of purpose of disease classifications is to guide treatment plans and predict patient prognosis based on a standardized framework. In the context of chronic pain, ICD-11 distinguishes between chronic primary and chronic secondary pain based on the underlying mechanisms and contributing factors. Chronic primary pain is characterized by a biopsychosocial model, where biological, psychological, and social factors are intertwined and play a significant role in the patient's pain experience (53). On the other hand, chronic secondary pain is typically associated with identifiable structural or pathological causes, such as overuse injuries seen in conditions like stiff shoulders or epicondylitis. However, it is important to acknowledge that psychosocial factors, such as stress, can also exacerbate pain in these conditions, even when the primary cause is physical (54).

In cases where pain persists or worsens after surgery or trauma, understanding the broader context—including the patient's history and psychosocial circumstances—is crucial (55). Although such cases are often classified as chronic secondary pain within the ICD-11 framework, integrating a biopsychosocial approach can offer a more comprehensive understanding and improve treatment outcomes (56). Even when the primary focus is on the structural cause, psychological factors can still significantly influence the patient's overall pain experience and response to treatment.

Another complex issue in applying this classification is evaluating the multifaceted psychological and social factors involved in chronic pain (57). In cases where an accident or lawsuit is associated with the onset of the condition, it can be difficult to disentangle the various contributing factors, such as the physical injury, psychological stress, and social circumstances. Rather than focusing solely on identifying a single cause, it is essential to recognize that chronic pain is often the result of multiple interacting factors. Additionally, the classification process may reflect clinical realities, such as the high comorbidity of chronic pain with psychiatric disorders and its higher prevalence among the elderly, rather than purely the subjectivity of the attending physician. The frequent classification of patients with psychiatric disorders, developmental disorders, and dementia as having chronic primary pain is likely indicative of these complex interactions (58).

Given that pain is inherently subjective and that pain intensity does not always closely correlate with pain-related disability, it is important to carefully consider how chronic pain is assessed and classified. While subjective patient-reported outcomes remain critical, relying solely on them could overlook key aspects of pain behavior. This highlights the need to explore improved approaches for classifying and utilizing the ICD-11 framework in the management of chronic pain (59).

### 4.4 Limitations

As this is a single-center study, there are several limitations that should be acknowledged. First, the results may not be generalizable to all patients with chronic pain in Japan, as our sample is limited to those who sought treatment at a single institution. Second, cultural factors, such as the traditional Japanese mentality of not openly expressing pain, may have influenced our findings (60). This cultural context may differ significantly from those in other countries and multiethnic populations, potentially limiting the applicability of our results on a global scale. Third, our study relied on self-report questionnaires to assess pain severity, pain, disability of pain, anxiety, depression, and other psychological factors. Self-report measures are subject to response biases, such as social desirability and recall bias, which can affect the accuracy of the data collected (61). Fourth, the cross-sectional nature of the study limits our ability to draw causal inferences. Longitudinal studies are needed to better understand the temporal relationships between psychological factors and chronic pain. Fifth, while our sample size provided sufficient power for initial analyses, larger sample sizes are necessary to confirm these findings and allow for more detailed subgroup analyses. A larger sample would also enhance the robustness of the conclusions drawn from our research (62).

Future research is necessary to consolidate information from interdisciplinary pain centers throughout Japan to provide a more comprehensive understanding of chronic pain across different regions and settings. Additionally, comparative studies involving patients from other countries and diverse ethnic backgrounds are essential to clarify differences and similarities in pain expression, management, and outcomes. Such studies will help to address the potential biases introduced by cultural factors and provide insights into the global applicability of our findings.

Furthermore, multi-center studies with diverse populations are needed to validate our results and enhance the robustness of the conclusions drawn from our research.

## 5 Conclusion

For the first time in Japan, patients with chronic pain were classified using ICD-11 codes, and their characteristics were analyzed. When comparing primary and chronic secondary pain, it was found that difficulties due to physical disabilities were more severe in chronic secondary pain, while psychological factors were more pronounced in chronic primary pain. A specific study of chronic primary pain grouped under MG30.01, which includes conditions like fibromyalgia, showed that patients classified as MG30.01 experienced greater physical difficulties, psychological factors including depression and anxiety, and had lower self-efficacy and quality of life compared to other chronic primary pain patients.

Although there are challenges in the classification process, it is anticipated that this classification will generate statistical information that will improve future medical care. Future research should focus on validating these findings across multiple centers and exploring the underlying reasons for the observed differences. Additionally, efforts should be made to refine the classification process to ensure consistency and reliability across different clinical settings.

## Data Availability

The raw data supporting the conclusions of this article will be made available by the authors, without undue reservation.
